# The Effect of Fascial Closure With Triclosan-Coated Sutures on the Incidence of Abdominal Wall Dehiscence: An Individual Participant Data Meta-Analysis 

**DOI:** 10.3389/jaws.2024.13337

**Published:** 2024-09-18

**Authors:** Allard S. Timmer, Niels Wolfhagen, Frank Pianka, Phillip Knebel, Christoph Justinger, Christos Stravodimos, Kosuke Ichida, Toshiki Rikiyama, József Baracs, András Vereczkei, Luca Gianotti, Marta Sandini, Jaime Ruiz-Tovar, Artur Marc-Hernández, Toru Nakamura, Marcel G. W. Dijkgraaf, Marja A. Boermeester, Stijn W. de Jonge

**Affiliations:** ^1^ Department of Surgery, Amsterdam UMC Location University of Amsterdam, Amsterdam, Netherlands; ^2^ Amsterdam Gastroenterology, Endocrinology and Metabolism, Amsterdam, Netherlands; ^3^ Department of General, Visceral and Transplantation Surgery, Heidelberg University Hospital, Heidelberg, Germany; ^4^ Department of Surgery, Marienhaus Klinikum Hetzelstift, Neustadt an der Weinstrasse, Germany; ^5^ Department of Surgery, Albert-Ludwigs University Freiburg, Freiburg, Germany; ^6^ Department of Surgery, Städtisches Klinikum Karlsruhe, Karlsruhe, Germany; ^7^ Department of Surgery, Saitama Medical Center, Jichi Medical University, Saitama, Japan; ^8^ Department of Surgery, Clinical Center, University of Pécs, Pécs, Hungary; ^9^ Department of Surgery, School of Medicine and Surgery, IRCCS San Gerardo Hospital, Milano-Bicocca University, Monza, Italy; ^10^ Department of Medical, Surgical, and Neurologic Sciences, University of Siena, Surgical Oncology Unit, Policlinico Le Scotte, Siena, Italy; ^11^ Department of Surgery, Rey Juan Carlos University, Madrid, Spain; ^12^ Department of Humanities and Social Sciences, University Isabel I, Burgos, Spain; ^13^ Department of Gastroenterological Surgery II, Hokkaido University Faculty of Medicine, Sapporo, Japan; ^14^ Amsterdam University Medical Center (UMC) Location University of Amsterdam, Epidemiology and Data Science, Amsterdam, Netherlands; ^15^ Amsterdam Public Health, Methodology, Amsterdam, Netherlands

**Keywords:** wound closure, fascial closure, fascial dehiscence, coated sutures, triclosan-coated sutures

## Abstract

**Introduction:**

Wound closure with triclosan-coated sutures (TCS) appears to reduce the risk of surgical site infection (SSI). Because there is a strong association between postoperative SSI and the development of acute abdominal wall dehiscence (AWD) after laparotomy, we hypothesized that the use of TCS for wound closure after laparotomy may also reduce the risk of AWD.

**Methods:**

The MEDLINE, Embase, and CENTRAL databases were searched from their inception to 01 November 2022. Randomized trials that compared the use of TCS with identical but uncoated sutures for fascial closure were eligible if they could provide individual participant data (IPD) on AWD. From these trials, we only included in the analysis those subjects who underwent open abdominal surgery. The primary outcome was the incidence of AWD within 30 days postoperatively, requiring emergency reoperation. The certainty of evidence was assessed using the GRADE methodology (PROSPERO: CRD42019121173.

**Results:**

We identified twelve eligible trials. Eight studies shared IPD. The incidence of AWD within 30 days after surgery was 27/1,565 (1.7%) in the TCS group vs. 40/1,430 (2.8%) in the control group (Relative Risk: 0.70 [95% confidence interval (CI) 0.44–1.11, *I* ^2^ = 0%, τ^2^ = 0.00]). The certainty of evidence was moderate after downgrading for imprecision. The incidence of incisional SSI was 163/1,576 (10.3%) vs. 198/1,439 (13.8%), RR 0.80 (95% CI 0.67–0.97).

**Conclusion:**

We found no conclusive evidence to support the use of triclosan-coated sutures for the prevention of acute abdominal wall dehiscence after laparotomy. In these selected studies, a significant reduction in incisional SSI was observed.

## Introduction

Acute abdominal wall dehiscence (AWD) or burst abdomen is a severe complication of open abdominal surgery. The reported incidence of AWD after laparotomy is as high as 3.8% [1–3]. It is associated with a high risk of mortality and generally requires emergency reoperation [4]. Patient characteristics such as age and pulmonary disease are associated with AWD, but the most important risk factor is surgical site infection (SSI) [4, 5].

Wound closure with triclosan-coated sutures (TCS) appears to reduce the risk of SSI [6–8]. Because this was not confirmed by a recently published large RCT, some controversy about the true effect of TCS on SSI remains [9]. Considering the association between SSI and AWD, we hypothesized that the use of TCS after open abdominal surgery may also reduce the risk of AWD. Two RCTs that primarily investigated the effect of TCS on SSI, reported a reduction in AWD. However, due to the low incidence of AWD and the relatively small sample size, uncertainty remains about the true effect [10, 11]. More data are needed to separate these findings from chance. Because of its major clinical consequences, AWD is generally well documented, and it is likely that existing trials have data on AWD available.

An individual participant data meta-analysis (IPDMA) is a meta-analysis of original trial data. Advantages of IPDMA include the possibility to include unpublished trial data, to in- or exclude individual participants, and to standardize the method of analysis to optimize statistical power. We aimed to investigate the effect of using TCS for wound closure after open abdominal surgery on the incidence of AWD using individual participant data from RCTs.

## Methods

### Study Registration

This study is registered in the International Prospective Register of Systematic Reviews (PROSPERO: CRD42019121173). Details of the protocol have been previously published [12]. We report according to the PRISMA-Individual Participant Data Statement [13].

### Eligibility Criteria

Randomized controlled trials that compared the use of TCS with the exact same but uncoated suture for wound closure (fascia, and possibly skin) after abdominal surgery were eligible for inclusion. Principal investigators of trials that reported only SSI incidence were contacted and asked if AWD incidence was registered and available. Trials were only included if the investigators could provide either IPD or aggregate data on the incidence of AWD. Exclusion criteria on the study level were prespecified and concerned trials that investigated the use of TCS as part of a bundle of interventions, trials that investigated the use of TCS after right lower quadrant incision for appendectomy, and trials that exclusively investigated children.

### Study Identification

The MEDLINE (PubMed), Embase (Ovid), and the Cochrane Central Register of Controlled Trials were searched with the aid of a clinical librarian. The complete search strategy is described in the Supplementary Material, page 2. The search was not restricted to publication date or language and was last updated on 01 November 2022. Backward citation tracking of previously published meta-analyses and included studies was performed. Furthermore, the International Clinical Trials Registry Platform was searched and corresponding authors of ongoing trials were asked if they were able to share unpublished data. In addition, all collaborating investigators were asked if they were aware of any other ongoing trials.

### Study Selection and Data Collection

Duplicate studies were removed and the titles and abstracts of the remaining studies were independently screened by two investigators. Subsequently, they retrieved and assessed the full texts of potentially eligible trials. The principal investigators of eligible trials were contacted. If the trial met the inclusion criteria, we invited the investigators to participate in the IPDMA. We proposed a set of data items with definitions that were finalized during an online collaborative meeting, as described in the study protocol [12]. Participating investigators were asked to de-identify the IPD before sharing. From the shared datasets, we only selected and analyzed participants who had undergone open abdominal surgery. The IPD from all studies was checked for missing, invalid, and out-of-range data, and for consistency compared to the published data. In case of concerns regarding data integrity we contacted the principal investigator. If significant issues could not be resolved after consultation with the principal investigator, the data were not included in the primary analysis.

### Risk of Bias Assessment

Two investigators independently assessed the risk of bias of the included trials using the Cochrane risk-of-bias tool for randomized trials (Rob2) [14]. The risk of bias was assessed using study protocols, published aggregated data, and shared IPD. Discrepancies were resolved through discussion. The presence of publication bias was assessed with a contour enhanced funnel plot [15].

### Outcomes

The primary outcome was the incidence of acute abdominal wall dehiscence (AWD), requiring emergency reoperation. Acute dehiscence was defined as spontaneous dehiscence of the abdominal fascia, with or without dehiscence of the skin, within 30 days after surgery. In case of reoperation for any indication other than AWD and when data were available, follow up continued until 30 days regardless of the suture used at the second procedure to avoid selective loss to follow up. The definition of AWD was established during a collaborative meeting and therefore universal across all trials.

The primary aim of our study is to investigate the effect of TCS on the risk of abdominal wall dehiscence. Study selection and all other aspects of the systematic review are based on this outcome and its availability. Secondary outcomes are merely exploratory and include incisional SSI within 30 days after surgery according to the Centers for Disease Control and prevention (CDC) criteria [16], skin dehiscence, length of hospital stay (days), all-cause reoperations, and all-cause mortality within 30 days after surgery.

### Missing Data

Missing data at participant level were imputed using multiple imputation by chained equations (MICE) for each trial separately [17, 18]. We performed five rounds of imputation and the number of missing data were recorded for each variable. If original trials imputed SSI, this imputed data set was used for imputation of AWD to ensure consistency and accuracy, as the original imputation had the most detailed data set available. Systematically missing data were not imputed.

### Data Analysis

Outcomes were analysed according to the intention-to-treat-principle in a one-step approach using a generalised linear mixed model framework. We accounted for clustering of participants within trials and potential baseline imbalances according to VanderWeele’s principles for confounder selection based and published literature on AWD risk factors [4, 5, 19]. Only variables that were available in all trials were eligible for confounder selection. We used a log-binomial model for binary outcomes and a linear regression model with bias corrected accelerated bootstrapping to estimate 95% confidence intervals (CI) for continuous outcomes with a non-normal data distribution. Treatment effects were expressed as relative risk (RR) and weighted mean differences (MD), with corresponding 95% CI for binary and continuous outcomes, respectively.

Several pre-specified additional analyses were performed. An as-treated analysis was done in which participants were analysed according to the actual suture that was used rather than the randomisation allocation. A per-protocol analysis was performed, according to the as-treated principle, and in which participants that underwent a reoperation for any reason other than AWD were excluded, because the suture - and thereby the intervention under investigation - was removed during a reoperation. Analyses were also conducted in a two-step approach. First, treatment effects were analysed for each trial. Subsequently, the aggregated data from all trials were analysed using a random-effects meta-analysis model (DerSimonian and Laird). Between study heterogeneity was assessed using the *I* ^2^ and Tau^2^ statistic (τ^2^).

We performed multiple sensitivity analyses on the primary outcome: a complete case analysis, midline incisions only, the use of TCS for both fascia and skin closure, studies that adequately blinded participants and personnel, exclusion of trials with high risk of bias, inclusion of all trials (regardless of data integrity concerns), and the addition of potential confounders that were not available in all trials and therefore not included in the primary analysis.

To explore heterogeneity and test for potential effect modification, pre-specified subgroup analyses were performed based on the specific type of suture (polyglactin 910 or polydioxanone), and wound classification according to the CDC criteria (CDC1 or CDC2-4) [16]. All analyses were performed using R version 4.0.3.

### Evidence Appraisal

We used the Grading of Recommendations Assessment Development and Evaluation (GRADE) working group methodology to evaluate the certainty of evidence for all outcomes [20]. As described in the study protocol, imprecision was evaluated using the optimal information size and the limits of the CI. Prior to data collection and analysis, we collaboratively defined a RR of 25% or more as clinically important threshold that would warrant rating down.

## Results

### Study Selection

**FIGURE 1:**
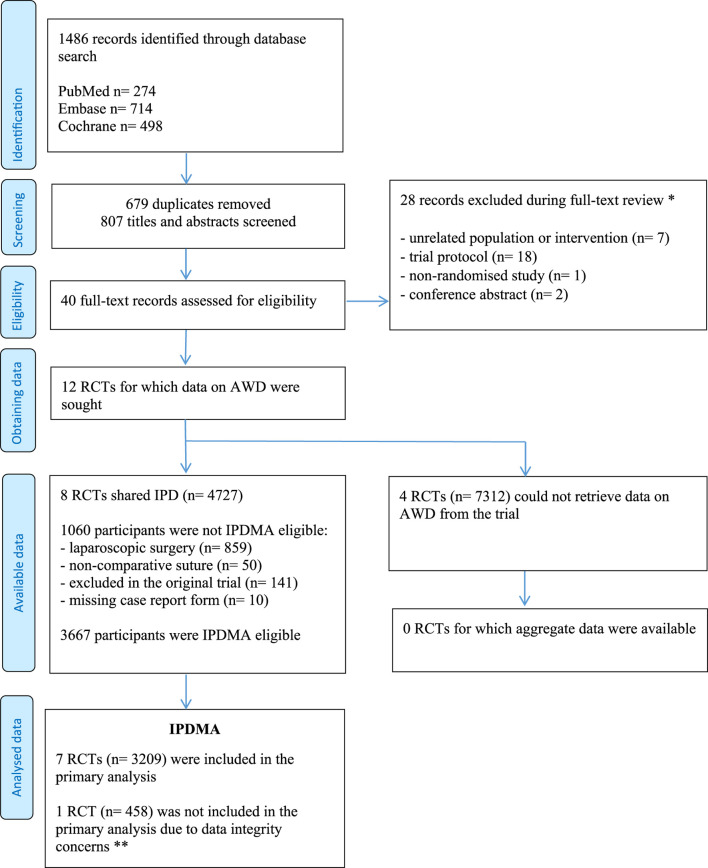
PRISMA Flow Diagram. * Reasons are shown in the Supplementary Material, page 3, ** A detailed description is provided in the Supplementary Material, page 6.

The study selection process is presented in Figure 1. The search strategy yielded 1,486 results. After full text assessment of 40 studies, 12 trials remained for which data on AWD were sought. The principal investigators from four trials (n = 7,312) declared that IPD on AWD from their trial was not available and could not be retrieved [9, 21–23]. The aggregate incidence of AWD was not reported in these trials. Eight trials shared IPD, of which two reported the incidence of AWD in their original publication [10, 11].

### Study Characteristics

Study characteristics of the eight trials are presented in Table 1. Trials were conducted in Europe (n = 6) and East Asia (n = 2) and published between 2011 and 2020. All trials used standardised preoperative antibiotic prophylaxis. Five trials [10, 11, 24–26] compared polydioxanone (PDS) Plus with PDS II and three trials [27–29] compared polyglactin 910 Plus with polyglactin 910, for closure of the abdominal fascia. Two trials [26, 27] also performed skin closure with TCS or control suture according to the same randomisation allocation. The primary outcome in all trials was SSI, assessed 30 days after surgery.

**TABLE 1:** Study characteristics of the included trials.

Study (year)	Country (no. of participating centers)	Surgery type	Intervention vs. control for fascial closure	Intervention vs. control for skin closure	Primary trial outcome	Eligible participants for IPDMA^a^
Diener et al. (2014)	Germany (24)	Elective midline laparotomy	PDS Plus vs. PDS II	n.a.	SSI^c^	1,185
Ichida et al. (2018)	Japan (1)	Gastroenterological surgery	Polyglactin Plus vs. polyglactin	PDS Plus vs. PDS II	SSI^c^	426
Justinger et al. (2013)	Germany/Switzerland (1)	Elective open abdominal surgery	PDS Plus vs. PDS II	n.a.	SSI^c^	967
Mattavelli et al. (2015)	Italy (4)	Elective clean-contaminated colorectal surgery	PDS Plus vs. PDS II	Polyglactin Plus vs. polyglactin	SSI^c^	246
Nakamura et al. (2013)	Japan (1)	Elective colorectal surgery	Polyglactin Plus vs. polyglactin	n.a.	SSI^c^	192
Ruiz-Tovar et al. (2015)	Spain (2)	Surgery with intra-operative diagnosis of fecal peritonitis	Polyglactin Plus vs. polyglactin	n.a.	SSI^c^	101
Ruiz-Tovar et al. (2020)	Spain (3)	Emergency surgery for infection or peritoneal contamination	PDS Plus vs. PDS II	n.a.	AWD and SSI^c^	92
Baracs et al (2011)^b^	Hungary (7)	Elective colorectal laparotomy	PDS Plus vs. PDS II	n.a.	SSI^d^	458

Abbreviations: TCS, triclosan-coated sutures; AWD, abdominal wall dehiscence; SSI, surgical site infection; PDS, polydioxanone, n.a. = not applicable.

^a^ Reasons for exclusion of participant: laparoscopic surgery (n = 859), use of a non-comparative suture (n = 50), excluded in the original trial (n = 141), missing case report form (n = 10) (see Figure 1).

^b^ Data from this trial were not included in the primary analysis due to unresolved data integrity concerns (Supplementary Material, page 6).

^c^ According to the Centers for Disease Control and Prevention criteria, within 30 days after surgery.

^d^ Assessed by telephone follow-up 30 days after discharge. Patients with wound related symptoms were invited for clinical assessment.

### Risk of Bias

Four trials [10, 27, 28, 29] including 2,049 participants were assessed at low risk of bias. One trial including 967 participants was assessed at moderate risk of bias due to the randomisation process [25]. Three trials including 651 participants could not share data of all randomised participants because of exclusion for reoperation, passing away or unknown reasons, and were therefore judged at high risk of bias [11, 24, 29] (Supplementary Material, page 4).

### Individual Participant Selection

Combined, the eight trials included 4,727 participants. We excluded 1,060 participants because they underwent a laparoscopic procedure (n = 859), received a non-comparative suture in a three-arm trial (n = 50) [11], were excluded from the original trial (n = 141), or had a missing case report form (n = 10). Exclusion on participant level was independent from randomisation allocation. One trial [25] excluded participants that were reoperated. Data of these participants were retrieved by the principal investigator and included in this study.

During the data verification process we detected possible integrity concerns in three trials. A detailed description of the integrity check is presented in Supplementary Material, page 5. Two trials were not registered before participant inclusion, and one of these contained a perfect distribution of operative indications across three trial arms [11, 29]. The principal investigator of these two trials provided us with the original study protocols, dated and signed before the trials had started. This included a description of a stratified randomisation that explained the distribution. Both trials were included in the primary analysis. In the third trial (n = 458) we detected discrepancies between the number of participants and the number of SSI between the IPD and the published data [24]. Because we were not able to resolve these differences with the principal investigator, data from this trial were excluded from the primary analysis but accounted for in a sensitivity analysis.

## Results of Synthesis

Summary results of the baseline and surgical characteristics are shown in Table 2. There were slightly more active smokers and patients with a previous midline incision in the TCS group, and slightly more patients with COPD in the control group. Other baseline characteristics where comparable between the two groups. The primary analysis contained 3,209 participants from seven trials; 1,670 allocated to the TCS group and 1,539 allocated to the control group.

**TABLE 2:** Baseline and surgical characteristics of the 3,209 participants from eight trials.

	TCS group (n = 1,670)	Control group (n = 1,539)
**Baseline characteristics**		
Age - years (mean, SD) missing not measured	65.0 (±12.3) 0 0	65.4 (±12.6) 0 0
Sex ratio – no M:F (ratio) missing not measured	1,023/647 (1.6) 0 0	954/585 (1.6) 0 0
ASA Physical Status score – no, (%) 1 2 3 4 missing not measured	130/1,544 (8.4) 890/1,544 (57.6) 506/1,544 (32.8) 18/1,544 (1.2) 31 95	140/1,353 (10.3) 790/1,353 (58.4) 411/1,353 (30.4) 12/1,353 (0.9) 88 98
BMI - kg/m^2^ (mean, SD) missing not measured	25.4 (±4.7) 27 95	25.2 (±4.7) 32 98
Active smoking– no, (%) missing not measured	245/1,009 (24.3) 7 654	205/1,018 (20.1) 15 506
Diabetes mellitus (any type) – no, (%) missing not measured	252/1,668 (15.1) 2 0	252/1,538 (16.4) 1 0
COPD – no, (%) missing not measured	83/988 (8.4) 0 682	113/1,006 (11.2) 2 531
Previous midline incision – no (%) missing not measured	159/680 (23.4) 0 990	148/696 (21.3) 1 842
**Surgical characteristics**		
Incision type – no, (%) Midline Non-midline missing not measured	1,553/1,670 (93.0) 117/1,670 (7.0) 0 0	1,443/1,539 (93.8) 96/1,539 (6.2) 0 0
Procedure type – no, (%) Elective Emergent missing not measured	1,572/1,670 (94.1) 98/1,670 (5.9) 0 0	1,432/1,539 (93.0) 107/1,539 (7.0) 0 0
Type of surgery – no, (%) Upper gastrointestinal Small intestine Colorectal HPB Other missing not measured	228/1,668 (13.7) 63/1,668 (3.8) 831/1,668 (49.8) 330/1,668 (19.8) 216/1,668 (12.9) 2 0	213/1,539 (13.8) 51/1,539 (3.3) 792/1,539 (51.5) 276/1,539 (17.9) 207/1,539 (13.5) 0 0
Wound classification (CDC criteria) – no, (%) 1 2 3 4 missing not measured	470/1,547 (30.4) 921/1,547 (59.5) 79/1,547 (5.1) 77/1,547 (5.0) 0 123	406/1,416 (28.7) 864/1,416 (61.0) 63/1,416 (4.4) 83/1,416 (5.9) 0 123
Duration of surgery – min (mean, SD) missing not measured	188 (±106) 7 218	192 (±106) 9 221

Abbreviations: CDC, centers for disease control and prevention; BMI, body mass index; COPD, chronic obstructive pulmonary disease; HPB, hepatopancreatobiliary.

Data are presented as mean and standard deviation (SD) or as count with percentage of the available data. *Missing* presents the number of participants with missing data at random. *Not measured* presents the number of participants for which a complete variable was not recorded in one or more trials.

Primary outcome results are presented in Table 3. Data on AWD were missing for 214 participants from two trials [10, 25]. For the remaining participants, the incidence of AWD within 30 days after surgery was 27/1,565 (1.7%) in the TCS group compared to 40/1,430 (2.8%) in the control group, resulting in a RR 0.70 [95% CI 0.44–1.11, *I* ^2^ = 0%, τ^2^ = 0.00].

**TABLE 3:** Primary and secondary outcomes.

	No. of studies analysed	Incidence in TCS group*	Incidence in control group*	Relative risk or mean difference (95% CI)**
**Primary outcome: AWD**				
Intention-to-treat missing	7	1.7% (27/1,565) 105	2.8% (40/1,430) 109	0.70 (0.44–1.11)^a^
As-treated^†^ missing	7	1.7% (26/1,562) 104	2.9% (41/1,432) 110	0.67 (0.41–1.07)^a^
Per-protocol^‡^ missing	7	1.5% (23/1,492) 42	2.9% (40/1,402) 52	**0.58 (0.34–0.99)** ^a^
**Secondary outcomes**				
Incisional SSI missing	7	10.3% (163/1,576) 94	13.8% (198/1,439) 100	**0.80 (0.67–0.97)** ^a^
Skin dehiscence missing	3	10.4% (83/797) 91	11.6% (94/810) 100	0.94 (0.69–1.27)^a^
Hospital stay (days)^§^ median, IQR missing	7	12 (9–19) 75	12 (9–17) 78	2.76 (2.72–2.81)^b^
All-cause reoperation^¶^ (30 days) missing	6	9.7% (158/1,623) 63	8.4% (125/1,490) 57	1.18 (0.93–1.48)^a^
All-cause mortality (30 days) missing	6	1.2% (19/1,625) 0	1.9% (28/1,491) 0	0.67 (0.38–1.19)^c^

* Based on crude IPD.

** Missing data at participant level were imputed.

^†^ According to the suture that was actually used during surgery. One participant received both TCS, and control suture and was excluded from the as-treated analysis.

^‡^ According to the as-treated principle and excluding participants that were reoperated for any reason other than AWD.

^§^ Presented as median and interquartile range and analysed using bootstrapping because of a non-normal data distribution.

^¶^ Three participants from one trial deceased within 30 days after surgery and data on reoperation were not available. These data were not imputed.

Variables included in the model:

^a^ gender, age, procedure type, type of surgery, diabetes mellitus

^b^ no adjustment

^c^ gender, age, procedure type, diabetes mellitus.

Bold indicates statistically significant results

The as-treated and per-protocol analyses resulted in a RR of 0.67 [95% CI 0.41–1.07], and 0.58 [95% CI 0.34–0.99], respectively. Results of the additional analyses are presented in the Supplementary Material, page 7. Sensitivity analyses showed similar results to the primary analysis. The addition of (slightly) imbalanced baseline variables that were not available in all trials did not significantly change the effect estimate in analyses of trials that had these variables available. Subgroup analyses showed no evidence of interaction between either suture type nor wound classification and treatment effect.

Secondary outcome results are presented in Table 3. The incidence of incisional SSI was 163/1,576 (10.3%) vs. 198/1,439 (13.8%), RR 0.80 (95% CI 0.67–0.97). Importantly, this is the pooled treatment effect from trials that were able to share data on AWD and not from all published trials investigating TCS. Additional analyses on SSI are presented in the Supplementary Material, page 8. The incidence of skin dehiscence [RR 0.94 (95% CI 0.69–1.27)] and all-cause mortality [RR 0.67 (95% CI 0.38–1.19)] were lower in the TCS group. Length of hospital stay was longer [MD 2.76 days (95% CI 2.72–2.81)], and incidence of all cause reoperation [RR 1.18 (95% CI 0.93–1.48)] were higher in the TCS group. The reasons for all-cause reoperation on participant level are presented in Supplementary Material, page 9.

### Certainty of Evidence

We only included RCTs, therefore the starting certainty of evidence was high. A contour-enhanced funnel plot did not indicate presence of publication bias (Supplementary Material, page 10). Trials with high risk of bias contributed less than 10% of the total data for the primary analysis. Furthermore, a sensitivity analysis without these trials provided a comparable relative risk [0.71 (95% CI 0.44–1.14)]. Overall risk of bias was therefore assessed as not serious. There was no evidence of serious inconsistency as statistical heterogeneity was low (*I* ^2^ = 0%). All trials provided IPD on the primary outcome; there was no indirectness. We assessed imprecision to be serious because the optimal information size was not met, and the CI overlapped appreciable benefit. Therefore, we downgraded the certainty of evidence with one level. As a result, the overall certainty of evidence for AWD was moderate (Supplementary Material, page 11).

## Discussion

In this individual participant data meta-analysis of 3,209 randomised participants, we found no conclusive evidence on the effectiveness of triclosan-coated sutures to reduce the risk of abdominal wall dehiscence after laparotomy, although a significant reduction in incisional SSI was observed. The certainty of evidence for AWD was moderate after downgrading for imprecision.

To date, only two RCTs have investigated the effect of TCS on AWD. Although both trials reported fewer AWD after the use of TCS, the findings were ascribed to chance as the sample size of both trials was based on an expected effect on SSI rather than AWD [10, 11]. As several meta-analyses show that TCS are effective in preventing SSI, their use has been conditionally recommended by the CDC, NICE, and the World Health Organization [30, 31]. Such effect of TCS on incidence of SSI was confirmed by the observations in the present study. Considering the strong association between SSI and AWD, these data support a potential biological mechanism for the effect of TCS on AWD [5]. However, contrary to our hypothesis, the combined data did not suffice to find conclusive evidence. We observed a considerably lower AWD incidence in the control group than the two trials that reported on AWD; 12.8% and 4.5% [10, 11]. This is likely attributable to the strict definition applied in our study. Only AWD that required reoperation were counted as event. The two previous trials applied a wider definition that also included AWD that did not require immediate reoperation and even asymptomatic AWD determined by radiologic imaging [10, 11]. Although a stricter definition with less events may impair statistical power and precision, it also minimises between-study variation and subjectivity in outcome definition. We deemed the latter more important.

It remains possible that clinically meaningful AWD - that did not require emergency reoperation but resulted in incisional hernias and long-term complications - were overlooked with the present approach. Long-term follow-up on the incidence of incisional hernia and reoperations may help understand the impact of TCS on these outcomes. Despite standardisation of the outcome, surgeons may still differ in their choice to perform a reoperation for AWD, potentially leading to bias. Because the main outcome of the trials was SSI, selective reoperation for AWD in any of the two groups is however unlikely. Furthermore, a sensitivity analysis investigating trials that adequately blinded participants and personnel for treatment allocation, was consistent with the primary analysis.

The cause of AWD is multifactorial. Technical errors such as tearing of sutures through the fascia, broken sutures after mishandling, or loose knots can result in AWD. Also, severe abdominal infection or ascites and postoperative coughing - leading to increased intra-abdominal pressure - or deep SSI are associated with AWD. On the other hand, it has been shown that fascial closure with small suture bites reduces the risk to develop an incisional hernia [32]. It could very well be that this specific technique also reduces the risk of AWD. Unfortunately, these data were not available for most of the trials. Due to inclusion of randomised data only, we have no reason to believe that disease severity or specific surgical techniques were imbalanced between the two arms and consider the observed effect independent of these aspects. However, it is unlikely that TCS use completely overcomes the risk of AWD due to gross technical errors or severely increased intra-abdominal pressure. Avoidance of AWD can only be achieved by addressing both patient and technical issues.

An unexpected finding is that all-cause reoperations occurred more frequently in the TCS group and hospital stay was slightly longer. We collected data on the reasons for reoperations to explore this difference, but they remained largely unknown because the two largest trials were unable to provide these data. Data from three smaller trials that could provide these data, revealed that all reoperations were performed for intra-abdominal complications unrelated to the suture used for fascial closure (Supplementary Material, page 9). This suggests that this difference may be a chance finding. Importantly, all-cause mortality was lower in the TCS group, suggesting that the difference in AWD was more consequential than reoperations for other reasons. Reoperation poses a further challenge in the interpretation of the data. Because the allocated suture - the intervention under investigation - is removed and the follow up of the wound resulting from the index operation is terminated. Whether these wounds would have healed without dehiscence had the intervention and follow up been left uninterrupted remains unknown.

In this individual participant data meta-analysis of randomised controlled trials, we found no conclusive evidence to support the use of triclosan-coated sutures for the prevention of abdominal wall dehiscence after laparotomy.
