# Supplementary material for: The Effect of Fascial Closure With Triclosan-Coated Sutures on the Incidence of Abdominal Wall Dehiscence: An Individual Participant Data Meta-Analysis
Source: J Abdom Wall Surg. 2024 Sep 18;3:13337. doi: 10.3389/jaws.2024.13337 (PMC11444969; doi:10.3389/jaws.2024.13337)
Supplement: Supplementary file 1 [file DataSheet1.docx]

**Supplementary Information**

**Manuscript Title**

The effect of fascial closure with triclosan-coated sutures on the incidence of abdominal wall dehiscence: an Individual Participant Data Meta-Analysis

**Table of content**

- Search strategy page 2
- Records excluded after full-text review page 3
- Risk of Bias assessment page 4
- Data integrity of individual trials page 5-6
- Additional analyses on AWD page 7
- Additional analyses on SSI page 8
- Reasons for all-cause reoperation page 9
- Contour enhanced funnel plot page 10
- GRADE assessment page 11

**Search strategy**

PubMed

("Triclosan"[Mesh] OR "Anti-Infective Agents, Local"[Mesh] OR triclosan*[tiab] OR antimicrobial*[tiab] OR antibacterial*[tiab] OR antiseptic*[tiab] OR antibiotic*[tiab]) AND ("Sutures"[Mesh] OR "Polyglactin 910"[Mesh] OR "Polydioxanone"[Mesh] OR suture*[tiab] OR vicryl*[tiab] OR polyglactin*[tiab] OR PDS II[tiab] OR polydioxanone*[tiab]) AND ("Surgical Wound Infection"[Mesh] OR "Surgical Wound Dehiscence"[Mesh] OR surgical wound infection*[tiab] OR surgical site infection*[tiab] OR postoperative infection*[tiab] OR surgical infection*[tiab] OR wound infection*[tiab] OR SSI[tiab] OR SSIs[tiab] OR abdominal wound dehiscence*[tiab] OR abdominal wall dehiscence*[tiab] OR fascial dehiscence*[tiab] OR burst abdomen*[tiab]) AND ("Randomized Controlled Trial" [Publication Type] OR "Controlled Clinical Trial" [Publication Type] OR "drug therapy" [Subheading] OR randomized [tiab] OR placebo[tiab] OR randomly[tiab] OR trial[tiab] OR groups[tiab]) NOT ("Animals"[Mesh] NOT "Humans"[Mesh])

EMBASE

(1) triclosan/ or exp topical antiinfective agent/ or (triclosan* or antimicrobial* or antibacterial* or antiseptic* or antibiotic*).ti,ab,kw. (2) exp suture/ or polyglactin/ or polydioxanone/ or absorbable suture/ or poliglecaprone suture/ or polydioxanone suture/ or polyglactin suture/ or (suture* or vicryl* or polyglactin* or PDS*).ti,ab,kw. (3) wound infection/ or surgical infection/ or wound dehiscence/ or (surgical wound infection* or surgical site infection* or postoperative infection* or surgical infection* or wound infection* or SSI or SSIs or abdominal wound dehiscence* or abdominal wall dehiscence* or fascial dehiscence* or burst abdomen*).ti,ab,kw. (4) randomized controlled trial/ or controlled clinical trial/ or drug therapy.fs. or (randomized or placebo or randomly or trial or groups).ti,ab,kw. (5) 1 and 2 and 3 and 4 (6) exp animal/ not human/ (7) 5 not 6 (8) limit 7 to conference abstract status (9) 7 not 8

Cochrane Central Register of Controlled Trials

(1) (triclosan* or antimicrobial* or antibacterial* or antiseptic* or antibiotic*):ti,ab,kw (2) MeSH descriptor: [Anti-Infective Agents, Local] explode all trees (3) #1 or #2 (4) (suture* or vicryl* or polyglactin* or polydioxanone or PDS*):ti,ab,kw (5) (surgical wound infection* or surgical site infection* or postoperative infection* or surgical infection* or wound infection* or SSI or SSIs or abdominal wound dehiscence* or abdominal wall dehiscence* or fascial dehiscence* or burst abdomen*):ti,ab,kw (6) #3 and #4 and #5 in Trial

**Records excluded after full-text review**

|  | **Author, year** | **Reason for exclusion** |
| --- | --- | --- |
| 1 | Ctri, 2017 | Study protocol |
| 2 | Ford, 2005 | Unrelated population (children) |
| 3 | Jprn, 2010 | Study protocol |
| 4 | Jprn, 2014 | Study protocol |
| 5 | Jprn, 2016 | Study protocol |
| 6 | Khachatryan, 2011 | Conference abstract |
| 7 | Mattavelli, 2011 | Study protocol |
| 8 | Mattavelli, 2013 | Study protocol |
| 9 | Matz, 2019 | Study protocol |
| 10 | McCallum, 2016 | Study protocol |
| 11 | Mingmalairak, 2009 | Unrelated population (appendectomy) |
| 12 | Nct, 2009 | Study protocol |
| 13 | Nct, 2009 | Study protocol |
| 14 | Nct, 2009 | Study protocol |
| 15 | Nct, 2010 | Study protocol |
| 16 | Nct, 2010 | Study protocol |
| 17 | Nct, 2012 | Study protocol |
| 18 | Nct, 2013 | Study protocol |
| 19 | Nct, 2018 | Study protocol |
| 20 | Nct, 2018 | Study protocol |
| 21 | Nct, 2020 | Study protocol |
| 22 | Okada, 2014 | Non-randomised study |
| 23 | Renko, 2017 | Unrelated population (children) |
| 24 | Roy, 2019 | Unrelated intervention (comparison of different sutures) |
| 25 | Serlo, 2016 | Unrelated population (children) |
| 26 | Tae, 2018 | Unrelated intervention (comparison of different sutures) |
| 27 | Yam, 2013 | Conference abstract |
| 28 | Zhuang, 2009 | Unrelated intervention (comparison of different sutures) |

**Risk of Bias assessment**

**
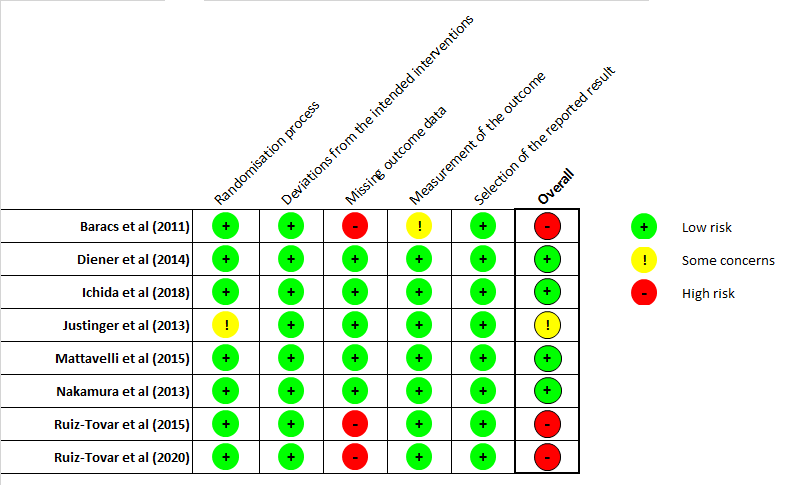
**

Two reviewers (AST and NW) independently assessed the risk of bias in the included studies using the Cochrane risk-of-bias tool for randomized trials. Study protocols, published aggregated data, and shared individual participant data were used.

**Data integrity check of individual trials**

The shared individual participant data were checked for missing, invalid/ and out-of-range values, and for notable or inconsistent results with the published trial data. Furthermore, when deemed necessary we requested original study protocols. During this process we encountered worth-mentioning issues in three trials.

Ruiz-Tovar et al. (2015) and Ruiz-Tovar et al. (2020)

These two trials have the same principal investigator. Both trials were registered on clinicaltrials.gov after inclusion of the first participant. The principal investigator declared to us that the trials were registered retrospectively because registration was a requirement for publication. For clarification, he sent us the original study protocols with corresponding ethical approval documents. Both contained adequate details of the methods section and were dated and signed before the start of the trials. Remarkable is that in both trials (n=110 and n=150) all eligible participants were willing to participate. The principal investigator ensured us that this was indeed correct.

Ruiz-Tovar et al. (2020)

The original registration on clinicaltrials.gov states SSI as primary outcome. In December 2019, after inclusion of the last participant the registration was amended, and evisceration was added as outcome data. We discussed this with the principal investigator who ensured us that evisceration had been an outcome of interest from the beginning of the trial. This was confirmed by the original study protocol.

Secondly, this trial included participants that were scheduled to undergo emergency surgery for four different infection-related indications. Table 2 of the manuscript states that the four indications for surgery are perfectly distributed over all treatment groups (this is a three-arm trial). This is remarkable because the manuscript does not mention a stratified randomisation process. We discussed this with the principal investigator who declared that the randomization was indeed stratified according to the indications for surgery, aiming to homogenise the sample. The original study protocol states that previous data from their hospital showed different rates of SSI for the different indications. Therefore, they had created a separate randomisation sequence for each pathology ensuring that the indications for surgery, with their own risk of SSI, were evenly distributed among treatment arms.

As such, there were no major unresolved concerns regarding the data from these trials. A sensitivity analysis excluding these two trials shows comparable results to the primary analysis (Supplementary appendix, page 7: *exclusion of trials with high risk of bias*).

Baracs et al. (2011)

When we compared the IPD with the aggregated trial data we observed a difference in the total number of participants (difference of ten) and in the number of surgical site infection (SSI) (difference of four). For both the IPD and aggregated data set, we created a table showing the total number of participants and the number SSI per randomization allocation. The IPD in the intervention group contained nine participants less and three SSI less. The IPD in the control group contained only one participant less and eight SSI less. We were not able to resolve these differences with the principal investigator of the trial. Therefore, the IPD from this trial were not included in the primary analysis. The effect of this decision was explored in a sensitivity analysis. (Supplementary appendix page 7: *All trials regardless of data integrity concerns*).

|  |  | **Intervention** | **Control** | **Total** |
| --- | --- | --- | --- | --- |
| **Aggregated data (publication)** | Participants (count) | 188 | 197 | 385 |
|  | SSI (count) | 23 | 24 | 47 |
| **IPD**  **(shared data)** | Participants (count) | 179 | 196 | 375 |
|  | SSI (count) | 20 | 16 | 36 |

| **Additional analyses on AWD** | | | | | | | | |
| --- | --- | --- | --- | --- | --- | --- | --- | --- |
| **Variable** | | **No of studies analyzed** | | **Incidence in TCS group*** | | **Incidence in control group*** | **Relative Risk (95% CI)**** |  |
| **Sensitivity analyses** | | | | | | | | |
| Two-step approach  missing | | 7 | | 1.7% (27/1565)  105 | | 2.8% (40/1430)  109 | 0.67 (0.44–1.04) |  |
| Complete case  missing | | 7 | | 1.7% (27/1565)  105 | | 2.8% (40/1430)  109 | 0.62 (0.39–1.00)^a^ |  |
| Midline incision only  missing | | 7 | | 1.7% (25/1448)  105 | | 3.0% (40/1334)  109 | 0.66 (0.41–1.06)^a^ |  |
| Use of TCS for both fascia and skin closure  missing | | 2 | | 1.8% (6/336)  0 | | 2.1% (7/336)  0 | 0.90 (0.30–2.57)^b^ |  |
| Trials blinding participants and personnel  missing | | 3 | | 1.5% (20/1359)    105 | | 2.6% (32/1219)  109 | 0.67 (0.40–1.14)^b^ |  |
| Exclusion of trials with high risk of bias  missing | | 5 | | 1.7% (25/1470)  105 | | 2.8% (37/1332)  109 | 0.71 (0.44-1.15)^a^ |  |
| All trials regardless of data integrity concerns  missing | | 8 | | 1.6% (28/1744)  105 | | 2.8% (40/1626)  109 | 0.70 (0.44 -1.11)^a^ |  |
| Adjustment for imbalanced baseline variables that were not available in all trials  (with and without COPD)  missing | | 5 | | 1.6% (12/884)  104 | | 3.0% (27/899)  109 | 0.60 (0.33–1.10)^a^  0.60 (0.32–1.10)^a^ | p-Wald: 0.60 |
| Adjustment for imbalanced baseline variables that were not available in all trials  (with and without Smoking)  missing | | 4 | | 1.6% (15/912)  104 | | 3.1% (29/924)  109 | 0.65 (0.37–1.15)^b^  0.65 (0.37–1.15)^b^ | p-Wald: 0.83 |
| Adjustment for imbalanced baseline variables that were not available in all trials  (with and without Previous Midline)  missing | | 2 | | 1.6% (9/576)  104 | | 3.7% (22/588)  109 | 0.59 (0.30–1.14)^b^  0.59 (0.30–1.15)^b^ | p-Wald: 0.94 |
| **Subgroup analyses** | | | | | | | | |
| **Subgroups** | | **No of studies analyzed** | | **Incidence in TCS group*** | | **Incidence in control group*** | **Relative Risk (95% CI)**** | **p-value of interaction** |
| PDS Plus vs PDS II  missing  Polyglactin Plus vs polyglactin  missing | | 4  3 | | 2.1% (25/1209)  105  0.6% (2/356)  0 | | 3.4% (36/1067)  109  1.1% (4/363)  0 | 0.70 (0.43–1.14)^c^  0.52 (0.09–2.80)^d^ | p=0.75 |
| Clean (CDC 1)  missing  Contamination (CDC 2-4)  missing | | 4  7 | | 1.4% (6/443)  27  1.9% (21/1122)  78 | | 1.6% (6/385)  21  3.0% (34/1045)  88 | 0.90 (0.32–2.56)^e^  0.66 (0.39–1.10)^a^ | p=0.64 |
| Abbreviations: *TCS*= triclosan-coated sutures, *AWD*= abdominal wound dehiscence, *PDS*= polydioxanone  * Based on crude IPD, ** Missing data at participant level were imputed  Variables included in the model: ^a^ age, gender, procedure type, type of surgery, diabetes mellitus; ^b^ gender, procedure type ^c^gender, age, procedure type, diabetes mellitus; ^d^ age, gender; ^e^ age, gender, procedure type, type of surgery | | | | | | | | |
| **Additional analysis on SSI** | | | | | | | | |
| **Variable** | **No of studies analyzed** | | **Incidence in TCS group*** | | **Incidence in control group*** | | **Relative Risk (95% CI)**** |  |
| **Sensitivity analyses** | | | | | | | | |
| Incisional SSI  missing | 7 | | 10.3% (163/1576)  94 | | 13.8% (198/1439)  100 | | 0.80 (0.67–0.97)^a^ |  |
| Superficial  missing  Deep  missing | 3  3 | | 11.2% (93/829)  94  4.1% (34/829)  94 | | 10.2% (85/834)  100  4.7% (39/834)  100 | | 1.08 (0.80–1.46)^a, ⴕ^  0.97 (0.62–1.51)^b,^ |  |
| **Subgroup analyses** | | | | | | | | |
|  | **No of studies analyzed** | | **Incidence in TCS group*** | | **Incidence in control group*** | | **Relative Risk**** | **p-value of interaction** |
| PDS Plus vs PDS  missing  Polyglactin Plus vs polyglactin  missing | 4  3 | | 10.8% (132/1220)  94  8.7% (31/356)    0 | | 14.3% (154/1076)  100  12.1% (44/363)    0 | | 0.82 (0.67–1.01)^a^  0.70 (0.46–1.07)^c^ | p= 0.54 |
| Clean (CDC 1)  missing  Contamination (CDC 2-4)  missing | 4  7 | | 6.5% (29/445)  25  11.8% (134/1131)  69 | | 10.8% (42/390)  16  14.9% (156/1049)  84 | | 0.64 (0.42–0.98)^c^  0.84 (0.69–1.04)^a^ | p= 0.26 |
| Abbreviations: *TCS*= triclosan-coated sutures, *SSI*= surgical site infection, *RR*= Relative risk, *MD*= Mean difference, *RR*= adjusted Relative Risk, *PDS*= polydioxanone  * Based on crude IPD, ** Missing data at participant level were imputed, ^ⴕ^ log-binomial model  Variables included into model: ^a^ age, gender, procedure type, type of surgery, diabetes mellitus; ^b^ age, gender, procedure type, diabetes mellitus ^c^ age, gender, diabetes mellitus | | | | | | | | |

**Reasons for all-cause reoperation on individual participant level**

| **Reoperation indication:** | **TCS** | **Control** |
| --- | --- | --- |
| Abdominal wound dehiscence* | 27 | 39** |
| Intra-abdominal bleeding | 3 | 2 |
| Bowel obstruction or perforation | 5 | 1 |
| Anastomotic leakage | 5 | 4 |
| Organ space SSI | 1 | 1 |
| Unknown | 118 | 80 |
|  |  |  |
| Total | 159 | 127 |

* From Ruiz-Tovar et all. 2015 data on all-cause reoperation were not available and data were not included for that outcome. Three participants from that trial developed an AWD that required reoperation. As such, the total number of all-cause reoperation presented in this explorative table shows a difference of three participants compared to the number presented in table 3.

** One participant in the control group developed an AWD for which reoperation was indicated but the participant refused surgery.

**Contour-enhanced funnel plot**

Although we included less than ten trials (the recommended minim to test funnel plot asymmetry) the contour-enhanced funnel plot of seven trials did not indicate presence of publication bias.

**GRADE assessment**

Question: Use of TCS compared to the exact same but uncoated suture for wound closure after open abdominal surgery

Setting: Randomised controlled trials

| **Certainty assessment** | | | | | | | **Certainty** |
| --- | --- | --- | --- | --- | --- | --- | --- |
| **№ of studies** | **Study design** | **Risk of bias** | **Inconsistency** | **Indirectness** | **Imprecision** | **Other considerations** |  |
| **Abdominal wound dehisce** | | | | | | | |
| 7 | randomised trials | not serious | not serious | not serious | serious | none | ⨁⨁⨁◯ Moderate |
| **Surgical site infection** | | | | | | | |
| 7 | randomised trials | not serious | serious | not serious | not serious | none | ⨁⨁⨁◯ Moderate |
| **Skin wound dehiscence** | | | | | | | |
| 3 | randomised trials | not serious | not serious | serious | serious | none | ⨁⨁◯◯ Low |
| **Hospital stay** | | | | | | | |
| 7 | randomised trials | not serious | serious | not serious | not serious | none | ⨁⨁⨁◯ Moderate |
| **All-cause reoperation** | | | | | | | |
| 6 | randomised trials | not serious | serious | not serious | not serious | none | ⨁⨁⨁◯ Moderate |
| **All-cause mortality** | | | | | | | |
| 6 | randomised trials | not serious | not serious | not serious | serious | none | ⨁⨁⨁◯ Moderate |
